# Genetic alterations of differentiated thyroid carcinoma in iodine‐rich and iodine‐deficient countries

**DOI:** 10.1002/cam4.781

**Published:** 2016-06-05

**Authors:** Huy Gia Vuong, Tetsuo Kondo, Naoki Oishi, Tadao Nakazawa, Kunio Mochizuki, Tomohiro Inoue, Ippei Tahara, Kazunari Kasai, Mitsuyoshi Hirokawa, Thong Minh Tran, Ryohei Katoh

**Affiliations:** ^1^ Department of PathologyUniversity of YamanashiYamanashiJapan; ^2^ Department of PathologyCho Ray HospitalHo Chi MinhVietnam; ^3^ Department of PathologyKuma HospitalKobeJapan

**Keywords:** BRAF, follicular, iodine intake, papillary, RAS, RET/PTC, thyroid cancer

## Abstract

*BRAF V600E* mutation, *RET* rearrangements, and *RAS* mutations are the common genetic alterations in differentiated thyroid carcinomas derived from follicular thyroid cells. However, the relationship between these alterations and iodine intake is still controversial. To clarify the influence of iodine intake on the occurrence of differentiated thyroid carcinomas, we performed molecular analyses for two differentiated carcinomas, papillary thyroid carcinomas (PTCs) and follicular thyroid carcinomas (FTCs), from an iodine‐rich country (Japan) and an iodine‐deficient country (Vietnam). We examined 120 PTCs (67 Japanese and 53 Vietnamese) and 74 FTCs (51 Japanese and 23 Vietnamese). We carried out allele‐specific polymerase chain reaction (AS‐PCR) for *BRAF V600E*, PCR and direct sequencing for *RAS* mutations (codon 12, 13, and 61 in *NRAS*,*HRAS,* and *KRAS*), and RT‐PCR for *RET/PTC1* and *RET/PTC3*. *BRAF V600E* was present in 55/67 (82.1%) Japanese PTCs and 44/53 (83%) Vietnamese PTCs. *RET/PTC1* was identified in only one PTC from each country, and no samples had *RET/PTC3*. *NRAS* mutation was found in 17/51 (33.3%) Japanese FTCs and 4/23 (17.4%) Vietnamese FTCs. *NRAS* mutation was cited in codon 61 (20 cases) and codon 12 (one case). None of FTCs had *KRAS* or *HRAS* mutations. There were no significant differences in the prevalence of *BRAF V600E*,*RET/PTC*, or *RAS* mutations between the two countries. Our study showed no differences in genetic alterations of thyroid cancers from iodine‐rich and iodine‐deficient countries, possibly suggesting that iodine intake might not affect the genetic alterations of differentiated thyroid cancer.

## Background

Iodine intake is considered a risk factor for thyroid carcinogenesis, especially in iodine‐deficient countries 1, 2. Experimental studies revealed that the rate of occurrence of thyroid carcinoma was higher in animals fed a low iodine diet than in animals fed a control diet 3, 4.

In 2005, the National Iodine Deficiency Disorders Control (NIDDC) Program reported Vietnam to achieve universal salt iodization and to eliminate iodine deficiency disorders. After the release of these national surveys, the Vietnamese government downgraded the NIDDC program from a national target to a routine program. This led to a critical decline, by 2009, of the national median urinary iodine concentration (UIC) to less than 83 μg/L, which was the lowest level of the last 10 years and below the optimal range of 100–199 μg/L recommended by the World Health Organization (WHO) 5. Interestingly, the median UIC in Ho Chi Minh City was only 56 μg/L 6. In contrast, Japan is regarded as a nongoitrous country due to regular consumption of iodine‐rich food. Zava and Zava 7 estimated that Japanese iodine intake, which comes largely from seaweed consumption, ranges from 1 to 3 mg/day. The mean or median UIC of Japanese residents ranges from 281 to 3300 μg/L in many regions of Japan 8, 9, 10, 11, 12, 13, 14.

There are three main categories of thyroid cancers arising from follicular thyroid cells: well‐differentiated, poorly‐differentiated, and undifferentiated. Papillary thyroid carcinoma (PTC) and follicular thyroid carcinoma (FTC) are well‐differentiated types with different genetic backgrounds.

Research in recent decades, has increased our understanding of the molecular pathogenesis of thyroid cancer. Among the genetic alterations involved in thyroid tumorigenesis, *BRAF* mutations, *RAS* mutations, and *RET* rearrangements are important in differentiated thyroid carcinomas. *BRAF* is serine–threonine kinase that is translocated to the cell membrane after being bound and activated by *RAS*. *BRAF* mutations are the most common mutation in PTCs, and the transverse point mutation at codon 600 (*BRAF V600E)* is the most common type of *BRAF* mutations 15, 16, 17. Several studies demonstrated a strong association between *BRAF* mutations and poor clinicopathological outcomes for patients with PTCs 18, 19, 20. *RAS* mutations can be found in follicular neoplasms and are more common in FTCs (40–50%) than in follicular thyroid adenomas (20–40%) 21, 22, 23, 24. There are three isoforms of *RAS* mutations: *NRAS, HRAS*, and *KRAS. NRAS* mutations occur predominantly in thyroid cancer 21, 25. *RET* is a proto‐oncogene encoding a RTK (receptor tyrosine kinase). There are more than 10 types of *RET/PTC* rearrangements, and the most common types are *RET/PTC1* and *RET/PTC3* 26.

The relationship between the occurrence of *BRAF V600E* or *RAS* mutations and iodine intake is unclear; there are only a few publications on this subject. Guan et al. 27 reported that the prevalence of the *BRAF V600E* mutation in PTCs was significantly higher in iodine‐rich areas than in iodine‐normal areas in China. Another study from Italy, however, did not find a statistical difference in the *BRAF V600E* mutation rate of PTCs between iodine‐rich and iodine‐deficient areas 20. The prevalence of *RAS* codon 61 mutations in FTCs was reported to be five times higher in the iodine‐deficient country of Hungary than in iodine‐rich Canada 28, and a possible relationship of iodine deficiency and generation of *RET/PTC* rearrangements has been suggested 29. However, Bartolone et al. 30 failed to detect *RAS* mutations in either iodine‐rich or iodine‐deficient FTCs.

The results from these studies demonstrate that there is a continued debate over whether iodine intake influences thyroid tumorigenesis. Therefore, we aimed to compare *BRAF V600E, RET/PTC* rearrangements (RET/PTC1 and RET/PTC3), and *RAS* mutations (*NRAS*,* HRAS,* and *KRAS*) in PTCs and FTCs from an iodine‐rich country (Japan) and an iodine‐deficient country (Vietnam) to clarify the relationship between iodine intake and genetic alterations of well‐differentiated thyroid carcinoma.

## Materials and Methods

### Patients and thyroid tissue samples

Our institutional review board approved this study, and all patients gave informed consent for collection of their data and tissue samples for this study. From 2011 to 2014, we randomly selected 67 patients and 53 patients with primary PTC who had been initially treated at University of Yamanashi Hospital (Yamanashi, Japan) and Cho Ray Hospital (Ho Chi Minh city, Vietnam), respectively. Between 2006 and 2014, we also selected 51 and 23 consecutive FTC cases from Kuma Hospital (Kobe, Japan) and Cho Ray Hospital, respectively. We confirmed the histopathological diagnosis of all cases based on WHO classifications 31. Retrospective review of surgical medical records provided patients' age and sex for all cases, plus prognostic information on tumor size, extrathyroidal extension, and any lymph node metastasis for the PTC cases. Clinicopathological data of the FTC cases such as tumor size, extrathyroidal extension, and lymph node metastasis were not available.

### DNA isolation and mutational analysis

Genomic DNA was extracted from formalin‐fixed paraffin‐embedded (FFPE) tissues using the RecoverAll^™^ Total Nucleic Acid Isolation Kit (Ambion, Austin, TX, US), according to the manufacturer's instruction. *BRAF V600E* in exon 15 of the *BRAF* gene is the most common point mutation detected in PTCs. As a result of this, we analyzed this mutation by conventional allele‐specific polymerase chain reaction (AS‐PCR) as described previously 32. Specific primers for *BRAF V600E* AS‐PCR amplify a 126‐base‐pair product using the primers described in Table 1 under the following conditions: (i) 94°C for 5 min; (ii) 40 cycles of 95°C for 15 sec, 60°C for 30 sec, and 72°C for 30 sec; (iii) 72°C for 10 min then (iv) hold at 4°C. Amplification of *BRAF* exon 15 was the quality control for extracted FFPE samples. *BRAF* exon 15 primers' designation and amplification protocol were performed as described previously 33. Human thyroid cancer cell lines KTC‐1 and WRO were the positive and negative controls, respectively, for *BRAF V600E* AS‐PCR. Electrophoresis for 30 min on agarose gel 3% (Agarose I^™^ Amresco, Solon, OH, US) and ethidium bromide staining allowed visualization of the PCR products.

**Table 1:** List of primers used for PCR

Primer	Size (bp)	Primer sequences (5′–3′)
*BRAF V600E*	126	Forward: GGTGATTTTGGTCTAGCTACATAReverse: GGCCAAAAATTTAATCAGTGGA
*RET/PTC1*		
Primary PCR	165	Forward: GCTGGAGACCTACAAACTGAReverse: GTTGCCTTGACCACTTTTC
Nested PCR	85	Forward: GCACTGCAGGAGGAGAACCReverse: CCAAGTTCTTCCGAGGGAAT
*RET/PTC3*		
Primary PCR	154	Forward: ACCTGCCAGTGGTTATCAAGCTReverse: TTCGCCTTCTCCTAGAGTTTTTCC
Nested PCR	80	Forward: CCAGGACTGGCTTACCCAAAReverse: CCAAGTTCTTCCGAGGGAAT
*NRAS*		
Codon 61	124	Forward: ACACCCCCAGGATTCTTACAGAReverse: GCCTGTCCTCATGTATTGGTC
Codon 12–13	132	Forward: TACTGTAGATGTGGCTCGCCReverse: CTGGATTGTCAGTGCGCTTT
*HRAS*		
codon 61	125	Forward: GGTGGTCATTGATGGGGAGAReverse: TGATGGCAAACACACACAGG
codon 12–13	156	Forward: TGAGCAGGGCCCTCCTTReverse: ATGGTTCTGGATCAGCTGGA
*KRAS*		
codon 61	129	Forward: TCCAGACTGTGTTTCTCCCTTReverse: TGTACTGGTCCCTCATTGCA
codon 12–13	117	Forward: GGCCTGCTGAAAATGACTGAReverse: TGTTGGATCATATTCGTCCACA

We detected mutations in the *NRAS, HRAS,* and *KRAS* genes of the FTC cases through direct sequencing of genomic DNA. PCR amplified exons 2 and 3 of each *RAS* gene followed by Big Dye terminator cycle‐sequencing reaction and sequence reading on an ABI PRISM 3130 genetic analyzer (Applied Biosystems, Foster City, CA). All primers for *NRAS, HRAS,* and *KRAS* exons 2 and 3 were designed with Primer3 software 34. Table 1 provides primer details.

### RNA extraction and reverse‐transcriptase polymerase chain reaction (RT‐PCR)

We reverse transcribed 15.4 μL of RNA from FFPE samples to complementary DNA (cDNA) with Superscript II reverse‐transcriptase (Life Technologies, Carlsbad, CA) and primed with oligo‐dT, random hexamers, and ribonuclease inhibitor (Taqman, Qiagen, Hilden, Germany). RT‐PCR protocol was performed as described previously 35. All cDNA products were submitted to amplification of glyceraldehyde‐3‐phosphate dehydrogenase *(GAPDH)* as quality control for RNA integrity. Only cDNA samples producing the *GAPDH* band underwent *RET*/*PTC1* and *RET/PTC3* amplification. The sequences of nucleotide primers for *RET/PTC1* and *RET/PTC3* and the primary and nested amplification protocols were designed as described previously 35. Our positive control was the human thyroid carcinoma cell line TPC‐1 containing *RET/PTC1*.

### Statistical analysis

Statistical analysis was performed with the SPSS software, version 18. Age at diagnosis and tumor size were reported as mean ± SD. We used Student's *t*‐test to compare patient ages and chi‐squared and Fisher's exact test to analyze the prevalence of *BRAF V600E* and *RAS* mutations and the *RET/PTC* rearrangements between the two cohorts. A *P*‐value less than 0.05 was considered a significant result.

## Results

### *BRAF V600E* and *RET/PTC* rearrangements in PTCs

The mean ages of the 67 Japanese and 53 Vietnamese patients were 49.3 years (range, 21–96) and 43.5 years (range, 20–72), respectively (*P* = 0.215). Female patients predominated in both countries accounting for 67.2% of Japanese and 77.4% of Vietnamese patients. The mean tumor sizes in Japanese and Vietnamese PTCs were 1.36 and 1.46 cm, respectively (*P* = 0.638). There were also no statistical differences in the rate of extrathyroidal extension and lymph node metastasis between the two PTC cohorts (data not shown).

**Figure 1:**
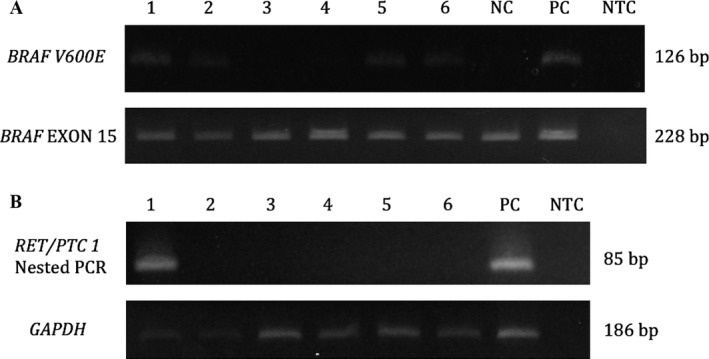
Representative results of (A) *BRAF V600E* mutation and (B) *RET/PTC1* in papillary thyroid carcinomas. *BRAF V600E* was detected in all but two samples (lanes 3 and 4). In contrast, only one sample had *RET/PTC1*. Please note concomitant *BRAF V600E* and *RET/PTC1* in a single sample (case 1). A. Lanes 1–6, samples from papillary thyroid carcinomas; NC, negative control (WRO cell line); PC, positive control (KTC‐1 cell line); NTC, no template control B. Lanes 1–6, same samples as Figure. 1A; PC, positive control (TPC‐1 cell line); NTC, no template control.

Allele‐specific PCR clearly demonstrated the band (126 bp) of *BRAF V600E* mutation in PTC samples that were microdissected from FFPE sections. Figure 1A shows representative results of the *BRAF V600E* mutation. The prevalence of *BRAF V600E* mutation in PTCs was rather high but similar in both countries; 82.1% in Japanese and 83.0% in Vietnamese patients. Table 2 shows the prevalence of *BRAF V600E* mutation in PTC histological variants.

**Table 2:** Histological types of papillary thyroid carcinomas and *BRAF V600E* status

Histology	No. of cases	Mutation status(%)			
		Japanese (*n* = 67)		Vietnamese (*n* = 53)	
		Negative	Positive	Negative	Positive
Classical type	110	9	55 (85.9)	6	40 (86.9)
Follicular variant	6	3	0	2	1
Tall cell variant	1	0	0	0	1
Solid variant	2	0	0	1	1
Macrofollicular variant	1	0	0	0	1

During RT‐PCR, we failed to extract RNA for *RET/PTC* amplification from FFPE sections in some PTC tissues. There were 62 of 67 Japanese samples (92%) and 51 of 53 Vietnamese samples (96%), respectively, available for *RET/PTC* amplification. Figure 1B shows representative results on *RET/PTC1*. We detected *RET/PTC1* in only one Japanese and one Vietnamese sample, and we detected no *RET/PTC3* in any PTC samples. A Japanese PTC positive for *RET/PTC1* concomitantly showed *BRAF V600E* mutation. Figures 1A and B, lane 1 (same case) show a dual positivity for *RET/PTC1* and *BRAF V600E* mutation.

### *RAS* mutations in FTCs

There was no statistical difference in mean patient age between the two countries; 54.2 years (range, 21–84) in 51 Japanese patients and 50.9 years (range, 22–75) in 23 Vietnamese patients (*P* = 0.289). The gender ratio of the FTC cases from the two countries was also similar (*P* = 0.903).

**Figure 2:**
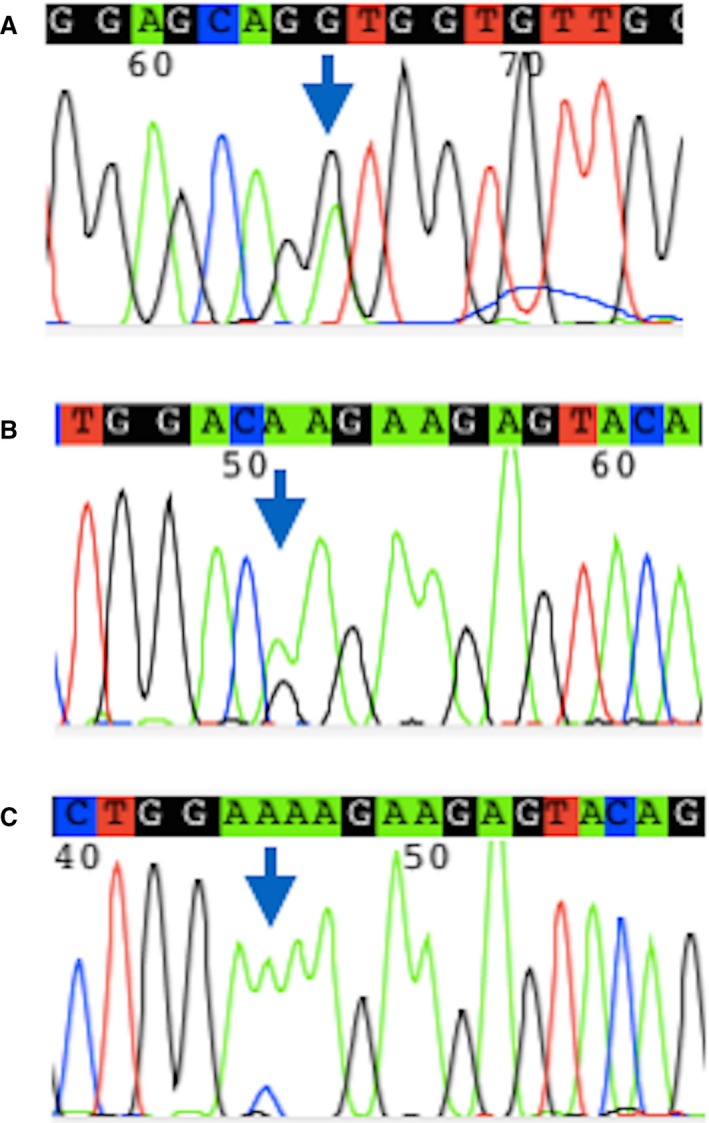
Representative results of *NRAS* mutations in follicular thyroid carcinomas A. *NRAS G12D**:*** G to A transition at nucleotide 35 (c.35G>A). B. *NRAS Q61R**:*** A to G substitution at nucleotide 182 (c.182A>G). C. *NRAS Q61K**:*** C to A substitution at nucleotide 181 (c.181C>A).

Point mutations were detected in *NRAS,* but there were none in *KRAS* or *HRAS*. The frequency of *NRAS* mutations in follicular thyroid carcinomas was 33.3% in Japanese cases and 17.4% in Vietnamese cases. Although the prevalence rate was two times higher in Japanese patients than in Vietnamese patients, this was not statistically different as measured by Fisher's exact test (*P* = 0.265). In *NRAS* mutations at codon 61, A to G substitution at nucleotide 182 was most common, and there was a C to A substitution at nucleotide 181 in one case. One Japanese FTC showed *NRAS* mutation at codon 12, which was a G to A substitution at nucleotide 35 (Fig. 2). Prevalence of *BRAF V600E, RET/PTC* rearrangements in PTCs, and *RAS* mutations in FTCs between two countries is summarized in Table 3.

**Table 3:** The prevalence rate of genetic alterations in Japanese and Vietnamese thyroid cancer

Genetic alterations	Mutated cases/total cases (%)		*P‐*value
	Japanese	Vietnamese	
*BRAF V600E* mutation in PTCs	55/67 (82.1)	44/53 (83.0)	0.89
*RET/PTC* rearrangements in PTCs	1/62 (1.6)	1/51 (2.0)	1.00
*RAS* mutations in FTCs	17/51 (33.3)	4/23 (17.4)	0.27

PTC, papillary thyroid carcinoma; FTC, follicular thyroid carcinoma.

## Discussion

Iodine is an essential element that plays a role in the synthesis of thyroid hormones. It is, however, still controversial whether iodine uptake from daily food intake influences the occurrence of thyroid disorders including hyperplasia, inflammation, and neoplasia. According to WHO data, Vietnam is an iodine‐deficient country with endemic goiter in up to 45% of the population in some regions 36. In contrast, Japan is an iodine‐rich country with high iodine intake, mostly from sea food consumption, especially seaweed 7.

This is the first study to investigate and compare genetic background (*BRAF V600E*,* RET/PTC* rearrangements, and *RAS* mutations) in differentiated thyroid carcinomas from iodine‐rich and iodine‐deficient countries. *BRAF* mutations are the most common mutations in PTCs, and *BRAF V600E* constitutes 99% of all *BRAF* mutations 37. The increase in prevalence of *BRAF* mutations in accordance with iodine supplementation and increase in proportion of PTCs have been reported in different geographical areas 38, 39, 40. This association led to a hypothesis that iodine intake may play a role, at least to some extent, in tumorigenesis of PTC and influence the increase of the *BRAF* mutations in PTCs. In this study, we found *BRAF V600E* in 82% of Japanese PTCs and in 83% of Vietnamese PTCs, which is not a statistically different prevalence rate for *BRAF V600E* (*P* = 0.894).

How iodine intake may influence *BRAF V600E* in PTCs is still controversial. Guan et al. 27 reported that high iodine intake was associated with a higher prevalence of the *BRAF V600E* mutation in Chinese PTCs. However, Frasca et al. 20 reported there was no statistical difference in *BRAF V600E* prevalence in Italian PTCs from an iodine‐sufficient area (39.6%) or iodine‐deficient area (34%) (*P* = 0.44). Although there are some differences in intrinsic and extrinsic factors, other than iodine intake, between Japanese and Vietnamese residents, our findings support a hypothesis that the amount of iodine consumption is not associated with the frequency of *BRAF V600E* in PTCs.

*RET/PTC* rearrangements appear almost exclusively in PTCs. There are many types of *RET* rearrangements, with *RET/PTC1* and *RET/PTC3* being the two most common types. Radiation‐induced PTCs commonly have *RET/PTC* rearrangements. An in vitro experiment demonstrated a dose‐dependent induction of *RET/PTC* in human thyroid cells following exposure to radiation 41. In an animal experimental study, Fiore et al. 42 reported that excess iodine acts as a protective factor in thyroid follicular cells during activation of *RET/PTC3* and attenuates the tumorigenesis process. To the best of our knowledge, this study is the first study comparing the prevalence of *RET/PTC* rearrangements in thyroid carcinomas between iodine‐rich and iodine‐deficient countries. In our study, the prevalence rate of *RET/PTC* rearrangements was low; *RET/PTC1* was detected in one sample from each country (1.6% of Japanese PTCs and 2.0% of Vietnamese PTCs), and *RET/PTC3* negative in all thyroid carcinomas. Our results suggested that *RET/PTC* rearrangements were not major genetic events of PTCs in both iodine‐rich and iodine‐deficient countries. The prevalence of *RET/PTC* rearrangements has been decreasing over the years 39, 43 which may explain the low frequency found in this study.

In follicular tumors, *RAS* mutations are the most common mutations and occur in 40–50% of FTCs 16. Iodine deficiency was considered a risk factor for thyroid cancer, particularly for FTC, following a comprehensive review of animal and human studies 44. Shi et al. 28 described the higher rate of *RAS* codon 61 mutation in an iodine‐deficient country. They concluded that *RAS* mutations may decrease with iodine intake, and that *RAS* oncogene activation might play a more important role in the initiation and/or maintenance of follicular tumors in iodine‐deficient countries. In our current study, we did not find any statistically significant difference in *RAS* mutations between iodine‐rich and iodine‐deficient FTCs. From these findings, we suggest that iodine consumption may not affect the *RAS* mutations in follicular cancer.

In conclusion, our study showed that there were no differences in genetic alterations of differentiated thyroid carcinomas from iodine‐rich and iodine‐deficient countries, possibly suggesting that iodine intake might not affect the genetic alterations of differentiated thyroid cancer.

## Conflicts of Interest

None declared.
